# ERP evidence for different strategies in the processing of case markers in native speakers and non-native learners

**DOI:** 10.1186/1471-2202-8-18

**Published:** 2007-03-02

**Authors:** Jutta L Mueller, Masako Hirotani, Angela D Friederici

**Affiliations:** 1 Neuropsychology Department, Max Planck Institute for Human Cognitive and Brain Sciences,, Leipzig, Germany

## Abstract

**Background:**

The present experiments were designed to test how the linguistic feature of case is processed in Japanese by native and non-native listeners. We used a miniature version of Japanese as a model to compare sentence comprehension mechanisms in native speakers and non-native learners who had received training until they had mastered the system. In the first experiment we auditorily presented native Japanese speakers with sentences containing incorrect double nominatives and incorrect double accusatives, and with correct sentences. In the second experiment we tested trained non-natives with the same material. Based on previous research in German we expected an N400-P600 biphasic ERP response with specific modulations depending on the violated case and whether the listeners were native or non-native.

**Results:**

For native Japanese participants the general ERP response to the case violations was an N400-P600 pattern. Double accusatives led to an additional enhancement of the P600 amplitude. For the learners a native-like P600 was present for double accusatives and for double nominatives. The additional negativity, however, was present in learners only for double nominative violations, and it was characterized by a different topographical distribution.

**Conclusion:**

The results indicate that native listeners use case markers for thematic as well as syntactic structure building during incremental sentence interpretation. The modulation of the P600 component for double accusatives possibly reflects case specific syntactic restrictions in Japanese. For adult language learners later processes, as reflected in the P600, seem to be more native-like compared to earlier processes. The anterior distribution of the negativity and its selective emergence for canonical sentences were taken to suggest that the non-native learners resorted to a rather formal processing strategy whereby they relied to a large degree on the phonologically salient nominative case marker.

## Background

Case marking is an important linguistic property shared by many languages in the world. In languages that allow free word order, such as Japanese and German, the correct interpretation of case markers is critical for sentence comprehension. For example, in Japanese the meanings of the sentences in (1) and (2) can only be distinguished by the correct interpretation of the nominative (nom.) and accusative (acc.) case markers, *ga *and *o*.

(1) 'Gakusei-*ga *kentikuka-*o *tasuketa.' (*Canonical*)

Student (*nom*.) architect (*acc*.) helped

*A student helped an architect*.

(2) 'Gakusei-*o *kentikuka-*ga *tasuketa.' (*non-canonical*)

Student (*acc*.) architect (*nom*.) helped

*An architect helped a student*.

While previous psycho- and neurolinguistic studies have shed some light on how case markers are used during online sentence comprehension in a native language (L1) [1-6], not much is known about potential difficulties confronting second language (L2) learners, or about differences between L1 and L2 speakers when case information is processed. With the present study we aim to contribute to the understanding of the neural underpinnings of the on-line use of case markers in native and non-native language processing. Therefore we examined both native speakers of Japanese and non-native learners whose L1 is German. As case is crucial in the present study, we will provide a short description of the case system in German and Japanese. We will focus on nominative and accusative cases as these were targeted in the present work. In German, case is encoded via an article that appears before a noun. The noun suffix changes its form according to the assigned case, grammatical gender and number. Although nominative-accusative-verb is the default (i.e., canonical) word order, the order of the case marked arguments is relatively free in German. Thus, the examples in (3) and (4) are both grammatical and have the same meaning.

(3) ...dass *der *Student *den *Architekten unterstützt.

...that the student (*nom*.) the architect (*acc*.) supports

...*that the student supports the architect*.

(4) ...dass *den *Architekten *der *Student unterstützt.

...that the architect (*acc*.) the student (*nom*.) supports

...*that the student supports the architect*.

In Japanese, case markers appear at the end of a noun phrase. As shown in (1) and (2), the nominative case marker *ga *is attached to the end of the subject of the sentence, and an accusative case marker *o *to the end of the object of the sentence. Unlike German, case agreement does not exist in Japanese. Therefore, nouns do not change their form according to the case markers assigned to the nouns. As for the basic word order, Japanese behaves like German (nominative-accusative-verb), and the order of the case marked elements is relatively free [7].

Event related potentials (ERPs) are an excellent tool for assessing neurochronometric aspects of language processing. Many studies using this methodology have linked specific ERP responses to different types of linguistic processes across different languages. Kutas and Hillyard were the first to report an ERP component, which was related to language processing. They discovered that semantically anomalous words within a sentence context elicited a centro-parietally distributed negativity peaking about 400 ms, which they termed N400 [8,9]. Much subsequent research has shown that the amplitude of the N400 increases together with lexical-semantic difficulties in a variety of contexts, such as word-lists, sentences and discourse context. It is a widely held view that the N400 reflects the semantic integration of a word into its wider context [10,11]. In the domain of syntax, a different group of ERP responses seems to be modulated. Two components which were frequently reported are left-anterior negativities (E/LANs) between 100 and 500 ms after stimulus onset [12-15], and P600 effects, which occur later (500–900 ms) over centro-parietal electrode sites [12,16,17]. It has been extensively debated as to which degree those components reflect specific syntactic processes [11,17] or rather domain-general cognitive mechanisms [14]. However, there seems to be considerable consistency in the conditions, which reliably elicit anterior negativities and/or P600 effects. While the LAN component seems to occur in response to outright (morpho)syntactic violations [14,15,18], the elicitation of a P600 does not require a syntactic error. It also occurs with syntactically complex [19,20] or ambiguous sentences [17,21]. The P600 can co-occur with both the N400 [22,23] and with left anterior negativities [14,15,18]. Thus, the LAN has frequently been related to morphosyntactic processing [11], and the P600 to later syntactic processes which could be syntactic reanalyses, repair [15,17] or processing of syntactic complexity [19]. Further, alternative accounts of these components will be mentioned later in the discussion of specific results. In studies investigating case processing, all of the abovementioned ERP components have been reported in specific paradigms. In the following we will review important findings of studies using the violation paradigm, with a specific focus on double case violations, which we chose to use as test cases for our experimental questions.

Starting with English, Coulson and colleagues [14] reported an ERP study in which participants read sentences containing case violations on a personal pronoun (e.g. '*The plane took we to paradise and back.' vs. 'The plane took us to paradise and back.'). Compared to the condition with correct case marking, a biphasic pattern, consisting of a LAN and a subsequent P600, was found for the condition with incorrectly case marked pronouns. Their interpretation for the LAN effect is based on working memory demands incurred by the search for plausible referents in a discourse context [14]. Another view of the LAN effect relates this component more directly to morphosyntactic processes instead of working memory [11,15]. The evidence for such a view comes from experiments which successfully dissociated a long-lasting negative shift in response to high working memory load from a local LAN effect in response to morphosyntactic violations [24,25]. The subsequent P600 found by Coulson et al. [14] was interpreted as belonging to the family of P300 components, triggered by the processing of an unexpected event (i.e. parsing an ungrammatical sentence). Other researchers, however, have argued in favour of a more syntax-related interpretation of the P600, and provided evidence for a functional distinction from P300 effects. The P600, for example, was shown to differ in scalp topography and task sensitivity, and to have additive effects with the P300 when both a syntactic anomaly and an unexpected non-linguistic event were presented simultaneously [26]. Furthermore, a recent study reported a dissociation of the oddball-related P300 and the syntax-related P600 in patients with lesions of the basal ganglia [27]. Thus, the P600 seems to be sensitive to syntactic manipulations in a different way than the domain-general P300. It is questionable, however, if the P600 is specific to syntactic processes. Some recent studies, in which the P600 was found for semantic instead of syntactic violations, see it as a more general monitoring component reflecting a process of checking the veridicality of ones sentence analysis [28,29]. On the basis of a linguistic interpretation of the LAN and the P600 component, Schlesewsky and Bornkessel pointed out that the LAN in Coulson et al.'s study probably reflects the mismatch between the pronoun case and the case which is required by the specific structural position in the sentence [30]. Interestingly, a more recent study in Dutch and German in which the processing of incorrectly case marked pronouns was investigated reported only a P600 component and no LAN [31]. To explain the absence of a LAN effect, the authors refer to language specific differences (i.e. fixed word order in English vs. free word order in Dutch and German) and to differences in the stimulus material.

In contrast to English, languages with overt morphological case marking allow for a greater variety of case violations. Case violations have been tested in the context of verb-argument processing [32,33] as well as contexts that are independent of verb information [22,23,34,35]. Studies in which case ambiguities or incongruencies were resolved at the verb position yielded N400 effects [32,33], LAN effects [33] as well as P600 effects [33]. Depending on the type of violation or ambiguity and depending on the sentence constructions used, different ERP effects seem to be triggered. As we chose to focus on case processing independently from verb-argument structure we will focus on those studies, which tested case incongruencies independently from verb information.

Several studies in German and Japanese which tested the effect of incorrect repetitions of the same case marker on nouns in a given clause reported N400 effects [22,23,34,35]. In those studies, two nominative (or accusative or dative) case marked phrases occurred in one clause, which is illegal in both languages. Frisch and Schlesewsky proposed that the occurrence of an N400 effect in such a linguistic context may be due to a problem of thematic hierarchization, i.e., the hierarchical ordering of the arguments according to thematic features, rather than reflecting lexical-semantic processes [22,23]. Note that this is not the classic view of the N400 effect. Generally, an N400 is considered to be an indicator of difficulties in (lexical-)semantic integration [10]. However, given the manipulation adopted in the studies of Frisch and Schlesewsky, which was clearly morphosyntactic, it is reasonable to assume that an N400 occurs beyond lexical-semantic contexts. The proposed thematic nature of the N400 was demonstrated by showing that it only occurred when the case conflict could not be resolved by animacy information, which is considered to be an important cue for building thematic relationships [36]. Besides establishing the thematic nature of their N400 effect, Frisch and Schlesewsky further observed an interesting asymmetry between nominative and accusative case markers in response to double case violation conditions [23]. The double accusatives led to an amplitude enhancement for the N400, compared to double nominatives. Their interpretation for the observed effect is based on the claim that those two case markers differ in terms of thematic markedness in German. Finally, in the same studies the N400 was accompanied by a P600 [22,23,34,35], which was taken as an indicator of syntactic aspects of the processing difficulty. However, the notion that the P600 in response to thematic violations is purely syntactic in nature has been challenged by a set of studies in which thematic constraints of a verb modulated P600 effects [28,29,37-39]. For example, in the studies by Kuperberg et al., verbs, which required an animate agent, led to a P600 effect when they were preceded by an inanimate agent [37,38]. As this efffect was further modulated by semantic factors such as plausibility, the authors maintain the view that the P600 may reflect the engagement in syntactic processes, but postulate that it can be modulated by semantic factors [38]. The observation that the P600 in Frisch and Schlesewsky's study was not modulated by animacy information suggests that their P600 effect was elicited by syntactic incongruency and not by semantic or thematic information.

Considering all the studies above, syntactic and thematic processing of case information performed by native speakers takes place at a relatively early stage in sentence processing, as indicated by the elicitation of ERP responses, which both occur between 300 and 500 ms after the critical element [14,22,23,34,35]. Whether this holds with highly trained non-native learners of a language is an interesting open question. To our knowledge, it is unknown how efficiently morphological case markers can be used by second language learners. In recent years, many studies on sentence comprehension using ERPs have shown that comprehension mechanisms for L2 speakers are quite different from those of L1 speakers. The linguistic domain in which ERP patterns in L2 learners seem to differ most from those in L1 speakers is the syntactic one. This is supported by the finding that ERP components elicited by syntactic violations for native speakers (early anterior negativities and/or P600 effects) are frequently aberrant or even absent in non-native speakers [40-43]. Interestingly, the N400 effect can be observed even at a very early stage of L2 learning [44]. However, even in the domain of lexical-semantic processing, latency delays or amplitude reductions were observed for the N400 [40,41,45]. Both P600 as well as N400 effects for lexical-semantic processing seem to be affected by proficiency as well as age of acquisition [40,42,46]. It is not clear how the processing of case and thematic information fits into our current understanding of L2 learning. As discussed above, comprehending non-canonical sentences like (2) and (4) is not an easy task for L2 learners. Case processing requires correct mapping of case markers and their grammatical function (e.g., subject and object of a sentence). Thematic processing involves conceptual processes on a relatively abstract level, such as computing features of thematic roles like agent, patient and undergoer, and finally, the mapping between case marked phrases and thematic roles has to be executed correctly. Potentially, learners may have access to native-like thematic mapping processes only at a later stage of learning, if at all. There is independent evidence showing that morphosyntactic processes are notoriously difficult for second language learners [47,48]. Since thematic processes are to a large degree dependent on the correct analysis of morphosyntactic information (i.e. case markers), they may not be easily acquired by L2 learners.

The first ERP study, by Mueller et al., revealed that learners' case processing mechanisms may be impaired at the level of thematic hierarchization [35]. In this study, native speakers of Japanese and German participants who were highly trained in a miniature version of Japanese, termed Mini-Nihongo, were presented auditorily with correct and incorrect Japanese sentences. While the native Japanese speakers showed a biphasic N400-P600 response for double nominative case violations (cf. example (6)), non-native learners exhibited the P600 but lacked the N400 response. These results were taken to suggest that the learners' earlier processes of thematic hierarchizing were impaired. There were at least two possible sources for the impairment. The lack of an N400 could be either due to the absence of the respective thematic process or it could be due to a kind of floor effect, which can be conceived as the recruitment of an upper limit of processing resources for correctly case marked arguments. Regardless of which explanation is correct, it has become evident that L2 learners face difficulties in on-line case processing.

(5) Ichi wa no hato *ga *ni hiki no nezumi *o *oikakeru tokoro desu.

One pigeon (*nom*.) two mice (*acc*.) run after is about to

*One pigeon is about to follow two mice*.

(6) *Ichi wa no hato *ga *ni hiki no nezumi *ga* oikakeru tokoro desu.

* One pigeon(*nom*.) two mice(*nom*.) run after is about to

Within the same study, however, not only the ERP results but also accuracy rates in a grammaticality judgment task were diffferent between non-native and native speakers. Thus, it was unclear whether the learners would still show different ERP responses from those of L1 speakers once they had reached higher proficiency in the miniature language. Moreover, the canonicity of the sentences used in the study (cf. examples (5) and (6)) may have encouraged participants to use a strictly linear processing strategy, whereby they may have attended only to the occurrence of a specific case marker in a particular sentence position, without relating the case marker to any thematic role. With those concerns about the previous study, the present study was aimed to ensure that the learners had a higher level of proficiency, and to include both canonical and non-canonical sentence structures to avoid a potential linear processing strategy. With the present study we addressed the following questions. First, we wanted to determine native Japanese speakers' ERPs in response to case violations in canonical (double nominative) and non-canonical (double accusative) sentences. Specifically, we were interested to see if potential differences between the two sentence types would correspond to the findings in German which were reported by Frisch and Schlesewsky [22,23]. The second question targeted the availability of native-like processing mechanisms for non-native learners. Although we found indications for non-native case processing mechanisms in a previous study we conjectured that higher proficiency as well as greater variability of sentence structures could lead to the emergence of more native-like patterns. Furthermore, we intended to test if the linguistic differences between the two types of case violations would have a similar impact on the processes of natives and non-natives.

**Figure 1:**
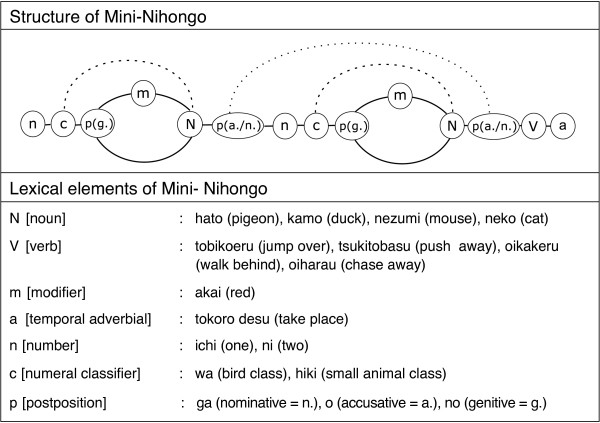
Schematic representation of structure and lexicon of Mini-Nihongo (illustration adapted from [35]). The nodes in the upper box denote word classes, solid lines mark legal transitions between elements of the classes, and dotted lines indicate dependencies between specific class members. Sentences are generated from left to right.

In the experiment we presented correct and incorrect canonical and non-canonical sentences taken from Mini-Nihongo (cf. Figure 1). The incorrect canonical sentences contained a double-nominative case violation, and the incorrect non-canonical sentences contained a double-accusative case violation (cf. Table 1). Two groups of participants were tested in the study. One was a group of native speakers of Japanese, the other one was a group of German participants who had received training until they were highly proficient in Mini-Nihongo.

## Results

### Behavioral Results

Both the Japanese and the trained groups reached high accuracy rates in the grammaticality judgment task for all conditions (see Table 2). The t-tests did not reveal significant differences between native Japanese speakers and trained non-native participants (p-values > .10).

### ERP Results

**Figure 2:**
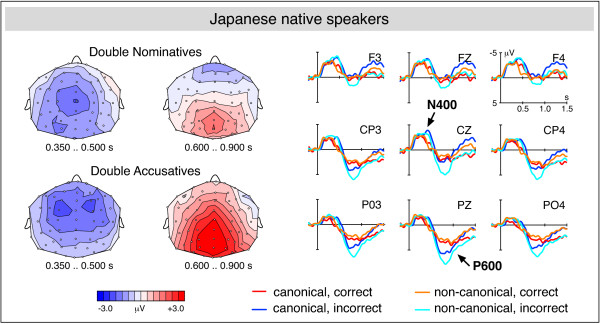
ERPs time-locked to the onset of the second noun for Japanese native speakers (red line: correctly case marked nouns in canonical sentences; orange line: correctly case marked noun in non-canonical sentences; dark blue line: double nominatives (canonical sentences); light blue line: double accusatives (non-canonical sentences). The topographical isovoltage maps represent difference potentials of the case violation minus the correct condition of the same sentence type.

**Figure 3:**
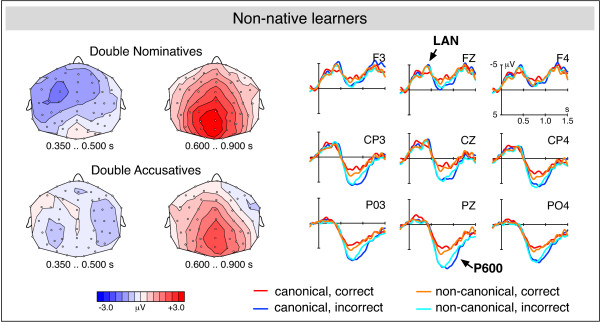
ERPs time-locked to the onset of the second noun for trained non-native participants (red: correctly case marked nouns in canonical sentences; orange: correctly case marked noun in non-canonical sentences; dark blue: double nominatives (canonical sentences); light blue line: double accusatives (non-canonical sentences). The topographical isovoltage maps represent difference potentials of the case violation minus the correct condition of the same sentence type.

Figure 2 and Figure 3 illustrate ERP-waveforms and isovoltage difference maps of the double nominative and double case violation conditions for native Japanese participants and non-native learners, respectively. The ERPs were time-locked to the onset of the second noun. As the plots illustrate, a negativity peaking at about 400 ms was found for double nominative case violations in both participant groups, while in the double accusative violation condition it seemed to be present only for native Japanese participants. In a later time window a posteriorily distributed positivity can be seen for the Japanese native and non-native groups for both double nominative case violations and double accusative case violations. While the positivity seems to have a larger amplitude for non-canonical sentences in Japanese native participants, this seems not to be the case for non-native participants. In the following we will present statistical results from ANOVAs in the two time windows from 350–500 ms and from 600–900 ms after stimulus onset. The main ANOVAs were calculated with unscaled data. In order to validate topographical differences we additionally report the significance level for the analysis with vector-scaled data for interactions which include a topographical factor.

### ERP Results: Negativity

The results of the omnibus ANOVA for both participant groups are summarized in Table 3. There was a highly significant main effect of the factor C. Furthermore, a significant interaction of G (native vs. non-native group) × C (correct vs. incorrect condition) × R (anterior vs. posterior region) × CA (non-canonical vs. canonical sentences) was found.

Subsequent analyses for each sentence type (canonical vs. non-canonical) separately revealed a significant interaction of R × CA × G for canonical sentences only (canonical: F(1,40) = 5.67, p < .05, scaled: p = .06; non-canonical: F(1,40) = 2.69, p = .11, scaled: p = .20). For non-canonical sentences a marginally significant interaction of C × G was found (F(1,40) = 2.97, p = .09), which was due to a significant main effect of C for the native group only (F(1,18) = 8.56, p < .01). Due to the significant interaction of R × CA × G for canonical sentences, separate analyses for each level group were done for those sentences. For the analyses of canonical sentences in the non-native group, a highly significant main effect of C (F(1,22) = 15.95, p < .001) as well as a marginally significant interaction of R × C was found (F(1,22) = 3.40, p = .08, scaled: p = .08). The native group showed only main effects of C (F(1,18) = 11.19, p < .01), but no significant interactions. As the only significant interaction effect which we found occurred for the non-natives, further analyses of simple main effects were done only for this condition and these sites. The simple main effects of C for non-native participants in the canonical sentence condition indicated a strong anterior distribution of the negativity (anterior: F(1,22) = 17.17, p < .001; posterior: F(1,22) = 5.02, p < .05).

In sum, the statistical analysis of the negativity between 350 and 500 ms revealed differences in occurrence and distribution of the effect. While Japanese native participants seemed to display a reliable negativity for both double nominative (canonical) and double accusative (non-canonical) conditions, non-natives showed a reliable negativity only for the double nominative condition. Moreover, the distribution of the effect appeared to differ across groups. Only non-natives showed a significant enhancement of the negativity over anterior electrode sites. Analyses with vector-scaled data which were additionally done when a topographic factor was included confirmed the analyses with unscaled data, suggesting that topographic differences were not only due to overall amplitude differences between the conditions.

### ERP Results: Positivity

The results of the overall ANOVA for trained non-native participants are illustrated in Table 3. The omnibus ANOVA revealed a main effect of C, and interactions at several levels including the 5-way interaction. The posterior distribution of the positivity was confirmed by subsequent analyses for each level of R separately (anterior: F<1; posterior:F (1,40) = 50.98, p < .0001).

Due to the significant 5-way interaction further ANOVAs were calculated for each level of CA. While the 5-way interaction could not be further confirmed (no reliable 4-way interaction was found for either level of CA), the interaction of C × G was significant for canonical sentences (F(1,18) = 4.55, p < .05) but not for non-canonical sentences. Further analyses of canonical sentences in each group separately revealed that there was no significant main effect of C for the native Japanese group. For non-natives, however, there was a significant main effect of C (F (1,22) = 15.78, p < .0001). To test if the 5-way interaction was driven by differences between canonical and non-canonical sentences, another 4-way ANOVA was calculated for each group separately including both levels of CA. The analysis for Japanese participants revealed a main effect of C (F(1,18) = 5.57, p < .05), an interaction of C × CA (F(1,18) = 10.08, p = .05) and an interaction of C × R (F(1,18) = 25.88, p < .0001, scaled:<.0001). The 4-way interaction was not significant for the Japanese group. Further tests at each level of CA revealed that there was a significant effect of C in the double accusative case violation (F(1,18) = 13.84, p < .01), but there was no significant effect of C for the double nominatives, as mentioned above. This difference was not driven by potential amplitude differences between correctly case marked nouns in canonical and non-canonical sentences (F<1), but rather by a significant effect of the factor CA for incorrectly case marked nouns (F(1,18) = 9.04, p < .01). The analyses of topographical distribution as indicated by the interaction of C × R confirmed the posterior distribution of the positivity (anterior: F(1,18) = <1; posterior:F(1,18) = 18.24, p < .001).

Further analyses for the non-native group revealed a significant main effect of C (F(1,22) = 15.79, p < .001), a significant two-way interaction of C × R (F (1,22) = 17.96, p < .001, scaled: p = .001), and a significant 4-way interaction of C × R × H × CA (F (1,22) = 9.25, p < .01, scaled: p = .01). As with the Japanese participants the C × R interaction was due to a posterior distribution of the main effect of C (anterior: F(1,22) = <1; posterior: F(1,22) = 18.24, p < .001) . To resolve the 4-way interaction, additional analyses of the simple main effects of C and of CA were done. Significant simple main effects of C were found for posterior electrode sites in both the canonical and the non-canonical conditions (canonical, post-left: F(1,22) = 42.75, p < .0001); canonical, post-right: F(1,22) = 22.93, p < .0001); non-canonical, post-left: F(1,22) = 8.23, p < .01); non-canonical, post-right: F(1,22) = 12.98, p < .01). The results of the analysis of simple main effects of CA indicate that there were (marginally) significant differences between the correct conditions (correct, ant-right: F(1,22) = 3.91, p = .06; correct, post-left: F(1,22) = 4.47, p < .05; correct, post-right: F(1,22) = 3.79, p = .06), but no differences between the incorrect conditions.

In sum, the statistical analysis for the time window between 600 and 900 ms confirmed a main effect of C which was modified by the other factors. The positivity was shown to be larger for incorrectly case marked nouns in the non-canonical condition compared to the canonical condition for Japanese native speakers. Non-native participants showed different modulations at the ROI level, which indicated that correctly case marked nouns in the canonical condition elicited a slightly more positive waveform, mainly over left posterior sites. In the canonical condition the overall amplitude of the positivity was larger for the non-native compared to the native group, while there was no difference in the non-canonical condition.

## Discussion

The present study tested how native and non-native listeners processed case marked arguments in canonical and non-canonical sentences of a miniature version of Japanese (Mini-Nihongo). Participants were native speakers of Japanese and a group of German native speakers who were trained up to high proficiency in Mini-Nihongo. Two types of case violations, double nominatives and double accusatives, were auditorily presented. Behavioural results did not reveal any differences between the groups. Nonetheless, ERP results suggested that the processing mechanisms across the two groups were not identical. Both participant groups showed a positivity between 600 and 900 ms for both violation conditions. For Japanese native speakers the effect was larger in the double accusative violation compared to the double nominative violation. Due to the linguistic nature of the task and due to its sensitivity to the different cases, we classify the positivity as a P600, reflecting syntactic processing mechanisms at the sentential level. A broadly distributed negativity was observed for both case violation conditions in Japanese native speakers. The learners, however, showed a similar effect only for the double nominative violation. Moreover, the learners' negativity was clearly enhanced over anterior electrode sites, which was not observed for Japanese native speakers in that condition. While the distribution of the effect as well as previous findings indicate that the effect is an instance of an N400 in Japanese native speakers, the strong anterior distribution in non-native listeners rather suggests a LAN component.

### Japanese native speakers

As expected, case violation conditions elicited a biphasic N400-P600 pattern when compared with non-violation conditions. This result is consistent with several previous experiments in different languages in which thematic processing costs were induced by violations of thematically relevant linguistic features. In English, which does not have overt morphological case marking, an N400-P600 pattern was found when verbs did not fit the thematic context of their arguments [37,38]. In languages with overt case marking, for example German and Japanese, double case violations elicited the same pattern [22,23,34,35]. Before turning to the interpretation of the specific modulations of these components that we found for Japanese native speakers, we will discuss the significance of the N400 and the P600 component in the context of thematic and case processing in more detail.

There are several studies in which thematically relevant semantic information modulated the N400 component. For example, Weckerly and Kutas [49] showed that inanimate subjects elicit a larger N400 amplitude compared to animate subjects, which are the more prototypical ones. Kuperberg and colleagues showed that thematic animacy violations on verbs elicited an N400 component [37,38]. While these authors maintain the classical view of the N400 as an index of semantic integration, Frisch and Schlesewsky [22] suggest that the N400 can also reflect a conflict which is genuinely thematic in nature. They draw this conclusion based on their study in which an N400 occurred in response to incorrect case markers with no other thematically relevant information available. There is further evidence that the N400 in the context of a problem in the thematic hierarchy may be neurophysiologically distinct from the N400 related to semantic integration difficulties. Roehm et al. [50] reanalysed the data of Frisch and Schlesewsky [22] using a frequency-band analysis. While the 'semantic' N400 (as measured in the comparison between animate and inanimate subjects) was characterized by evoked power in the upper theta frequency band, the 'thematic' N400 (as measured in the comparison of correctly vs. incorrectly case marked nouns) involved enhanced evoked power in the lower-theta frequency band. While these results clearly have to be validated by further investigations, they point to the possibility that N400 effects are a less unitary phenomenon than previously thought. If the N400 component in our experiment reflects difficulties in meaning integration or a more abstract conflict in the establishment of the thematic hierarchy of arguments cannot be determined easily. However, as there was no other information than case markers which induced the effect, we can conclude that the difficulty it reflects was induced by thematically relevant morphological information and not by lexical-semantic information.

As mentioned earlier, the P600, usually seen as a correlate of syntactic sentence comprehension mechanisms, has recently been showing up in response to semantic manipulations [28,29,37-39]. Kolk et al. (2003), for example, reported a P600 for semantic/thematic anomalies in sentences like '*The cat that fled from the mice ran across the room'. Such results clearly extend the range of conditions which elicit P600 components. It is not clear yet, however, if the processes reflected in the P600 are non-syntactic and general monitoring processes in these cases, as has been suggested by the authors [28,29]. It could still be possible that the semantic conflict is resolved by structural repair processes, for example by re-assigning subject and object status within a sentence. In our experiment the P600 could be induced by both, either by the syntactic difficulty induced by the formal incongruency between the case markers requested by the syntactic structure, or by thematic difficulties brought about by the incompatibility between thematic roles. Which information was crucial in our experiment cannot be decided unequivocally as we did not manipulate thematic and syntactic congruency independently. Aside from the uncertainty about the type of information eliciting the P600, we assume that the process itself which it reflects is related to increased syntactic processing costs afforded by the processing of the incongruency [11,17], although other views which regard the P600 as a more domain-general component are not inconsistent with our results [14,39]. In the following we will discuss the relevance of the present results for research on thematic and case processing across different languages. As an important addition to the study of Mueller et al. [35], the N400-P600 pattern was obtained not only for the double nominative condition but also for the double accusative condition. This leads us to conclude that in the Japanese language an N400-P600 pattern is associated with the processing of case conflicts in general and not with the processing of the particular nominative case marker or the specific thematic roles it assigns. Moreover, the present experiment revealed two interesting cross-linguistic differences. The first one is the observation that both double nominatives and double accusatives elicited a comparable N400 effect in Japanese while Frisch and Schlesewsky [23] reported an enhanced N400 for double accusatives in comparison to double nominatives in German [23]. With the assumption that accusative case requires a specific (dependent) thematic role (e.g., undergoer, patient), Frisch and Schlesewsky explain their finding in the following way: If more than one accusative case is present, more than one dependent thematic role is allocated to a clause. As the same thematic role cannot occur twice in an interpretable clause, a severe conflict is incurred during thematic processing. Since nominative case does not go along with a particular thematic role, the repetition of a nominative case marked argument does not necessarily lead to the repetition of the same thematic role. If such a relationship between nominative and accusative case and thematic role holds universally, a similar pattern of data could be expected for Japanese. But this is not what we observed. We did not find differences in the N400 amplitude for double nominative and double accusative violations for Japanese participants. We speculate that the difference comes from the materials used in the two studies. In their study, Frisch and Schlesewsky [23] tested an NP-V-NP structure. This contrasts with a verb final NP-NP-V structure in the present Japanese study. We conjecture that the prior presence of the verb induces a stronger thematic conflict as it adds even stronger thematic requirements to the upcoming arguments which are specified in its argument structure. As Japanese is a strictly verb final language the verb-argument-structure can impact thematic processes only at the end of a clause. It may well be the case that Japanese double accusatives are equipped with a greater conflict than double nominatives. However, without the early appearance of a verb, the associated conflict may not be as severe as the one in German, and thus not be reflected in modulations of the N400.

The second interesting difference from the results reported for German was that Japanese double accusatives elicited an enlarged P600, compared to double nominatives. We like to argue that the differences in the modulation of the P600 effect are due to linguistic differences of the case system between German and Japanese. In Japanese, but not in German, there is a specific linguistic constraint which could cause more substantial syntactic difficulties or more complex repair processes for the double accusative than for the double nominative violation. As formulated in the well-established *double-o constraint *[7], having a sequence of two accusative case marked phrases within the same clause is strictly prohibited in Japanese. Therefore, in a context in which no indication of a clause boundary between the two accusative case markers is provided (e.g., by prosody), the processor runs into a severe syntactic conflict upon entering the second accusative marker. As for double nominatives, Japanese technically allows two nominative case marked phrases to appear within the same clause, for example, when the phrase is focused or the verb is stative, e.g., *John-ga biiru-ga suki da *'John likes beer'. Despite this linguistic fact, however, it was previously pointed out that an analysis with double nominatives (within the same clause) occurs rather infrequently in readers' first pass [51,52], and hence, we believe this to be the source for the observed N400-P600 pattern. Previous studies testing the impact of different types of syntactic manipulations on the P600 component have shown that the P600 amplitude can be modified by the degree of syntactic incongruency, with clearly ungrammatical structures leading to larger amplitudes compared to non-preferred structures (e.g. [17]). In our case, the double accusative violation is clearly illegal while the double nominative violation becomes clearly illegal only at the verb position. At the position of the second nominative it is rather a less frequent structure. This difference may have resulted in a larger P600 effect for double accusative violations.

In sum, the present data provide cross-linguistic evidence for the on-line use of case markers in sentence comprehension (i.e., syntactic and thematic processing). With regard to native language processing, Japanese and German [22,23,34,35] share the overall pattern of ERPs for double case violations, namely an N400-P600 pattern. When it comes to differences between specific case markers, an interesting modulation was found in the ERP effects. Japanese, but not German, showed an enhanced P600 effect for the double accusative condition. This difference probably stems from the structural difference between the two languages as formalized in the *double-o constraint*.

### Trained non-native participants

The two types of case violations elicited distinct ERP patterns in the non-natives. Only in canonical sentences (double nominative) case violations elicited a negativity, however, with an anterior focus, while the P600 was present in both canonical and non-canonical sentences. While the amplitude of the P600 did not differ for incorrectly case marked nouns across cases, a slightly enhanced positivity was observed for correctly case marked nouns in the non-canonical condition.

The emergence of the negativity illustrates that the learners' processing system detects the incorrect nominative case marker *ga *in the same time window as the system of native speakers. Despite this similarity in timing, though, the topographical distribution of the negativity seems to be different. While it was broadly distributed in native Japanese speakers, it was anteriorily focused in the learners. For Japanese native speakers, the negativity was interpreted as an instance of an N400 reflecting processes of thematic hierarchizing. The topography of the negativity in the learners resembles much more the distributions of syntax related negativities, namely LAN components. As reviewed in the introduction, Coulson et al. reported a LAN component for a case violation in English when there was a case violation occurring on the pronoun [14].

Schlesewsky and Bornkessel interpret Coulson et al.'s results as being an indication of a 'mismatch between a particular structural position and the morphological realisation of the element encountered in that position' [30, p.1225]. According to this interpretation, participants did not use the pronoun case for thematic interpretation but rather relied on word order for this purpose. Thus, the morphosyntactic mismatch did not result in an N400, reflecting conceptual processes, but a LAN, reflecting the structural aspects of the mismatch. Is it possible that the learners in the present experiment resorted to a more formal, position-based strategy of case processing than the native speakers? Note that the experimental task did not require detailed thematic processing, as the grammaticality judgment could be given without fully comprehending the conceptual meaning of a sentence. One possibility would be that the learners used very simplified 'case-rules' such as 'if one case marker occurs in the first NP, a different one has to follow in the second'. In principle, such a rule would be sufficient and could potentially explain the more anterior distribution of the negativity, resembling the topography of LAN effects. (Left) anterior negativities have been rarely reported for L2 speakers [40–42, but see [53], experiment 1]. However, most studies reporting a missing left anterior negativity for L2 speakers tested word category processing [40-42], which is assumed to occur at an earlier processing stage than other morphosyntactic operations in 'syntax first' models of sentence comprehension [11]. The frequent absence of (left) anterior negativities in L2 speakers has been used to argue for the vulnerability of syntactic processes in L2 speakers at an early processing stage [40-42]. Some artificial grammar studies, on the other hand, seem to suggest that automatic syntactic processes can be acquired by adults if they are highly trained. Those artificial grammar studies, in which the tested syntactic operations were similar to syntactic operations in natural languages, revealed anterior negativities after relatively short training [54,55]. Thus, it seems to be the case that anterior negativities can occur in learners of a real or artificial language system, if the underlying formal operation is simple enough to process.

The occurrence of an anteriorily focused negativity in our case does not show that learners are syntactically more proficient than the native speakers. Rather, we see it as an indication that the learners process the case markers more superficially, restricting their processes to purely formal aspects, while native Japanese speakers use the case markers to establish a real thematic relationship between the arguments. With respect to vocabulary size (18 morphemes) and number of rules (7), Mini-Nihongo is very comparable to the stimuli used in artificial grammar studies in which anterior negativities have been reported for learners [54,55]. Thus, the small size of the miniature language may have freed resources of participants in order to process case information in the same time-window as native speakers, even though they may have resorted to a more formal strategy which lacked conceptual 'depth' of processing.

The simple application of more formal, less interpretive processes in the non-native participants does not explain, however, why only double nominatives led to a negativity. As participants were exposed to canonical and non-canonical sentence structures with equal frequency during learning, familiarity with a specific structure cannot account for the absence of the negativity for double accusative violations. Canonical and non-canonical sentences were not processed identically by the learners, though. In contrast to Japanese native speakers the non-natives exhibited an enhanced positivity for correctly case marked nouns in non-canonical sentences (which were in nominative case). This indicates that there was some additional processing with regard to non-canonical sentences in general, which may have interfered with the initial detection of the case violation. If we assume a relatively shallow processing strategy for the learners, it is not clear why non-canonical sentences should be more difficult for the learners. One possibility would be that the learners adopted a general subject-first strategy, as was postulated for many languages (e.g., [56]). An additional reason, however, why non-canonical sentences might be more difficult lies in differences in phonetic markedness between the nominative *ga *and the accusative *o *case marker. Note that nominative case is marked by a *ga *postposition (e.g., hato *ga*), while accusative case is marked by an *o *postposition (e.g., hato *o*). In the case of an accusative case marked noun, two consecutive vowels have to be segmented from each other, while in the case of a nominative case marked noun, a stop consonant has to be segmented from the preceding vowel. The possibility that L2 learners are prone to difficulties in discriminating vowel-vowel transitions is also supported by the finding of Nenonen et al. [57], who reported difficulties in L2 speakers in the processing of phoneme contrasts based on vowel length, as reflected in the modulation of the Mismatch Negativity. Similarly, Frenck-Mestre and colleagues reported modulations of ERP responses for a non-native vowel placed in an environment in which it was difficult to perceive [58]. Indication that early processes of word segmentation can be difficult in general for L2 speakers stems from a study of Sanders and Neville [59], where they showed that Japanese second language learners of English lacked an N100 effect in response to word initial compared to word medial syllables. Similarly to these studies, in our experiment the spectral changes which mark the transition from a vowel to the stop consonant g may be more salient and easier to perceive for the trained participants, compared to the vowel-vowel transition. In three of four cases of accusative case marked noun phrases in the present experiment, the vowel-vowel transition even consists of a sequential repetition of the same vowel *o*, which may be similarly difficult to distinguish, as vowel-length based phoneme contrasts within words. If the participants used a strategy in which they relied only on the more salient case marker, the absence of the negativity for double accusatives as well as the increased positivity for correctly case marked nouns in non-canonical sentences could be explained. In this scenario listeners would not assign case if a sentence starts with an accusative case marked phrase. Instead, they wait until a nominative (and salient) case marked noun appears. An enhanced positivity for the second NP in scrambled sentences could therefore indicate additional processing costs related to the retrospective assignment of case. In canonical sentences participants can use the nominative case marker earlier, and thus can build a syntactic structure already from the beginning of the sentence.

Note that this supposed strategy did not go along with behavioural difficulties. As we used a delayed response task, leaving the participants an ample amount of time to take their decision, it is possible that this result is in fact a ceiling effect. However, it is also conceivable that the strategy that was applied by the participants was equally efficient in solving the task as the strategy applied by native speakers. We would like to point out that the high level of performance should not be taken as proof of native-like proficiency, but rather as an indication of very high task specific skills in the restricted system of Mini-Nihongo. One could see the absence of a difference in the P600 amplitude between native and non-native speakers in a similar way. Most studies on L2 processing reported P600 differences with reduced amplitudes for non-native speakers [42,43,60]. In our study the P600 was even larger for non-natives compared to natives in the double nominative condition. First, the absence of a difference between natives and non-natives lets us assume that the extensive training provided the learners with sufficient input to develop a native-like P600. Age-of-acquisition or proficiency effects, which are otherwise frequently observed for non-native speakers, may thus be overridden by the very high task-specific skills. Second, the enhanced amplitude for canonical sentences is not surprising when we consider that the learners did not learn about potentially grammatical double nominative constructions during their training. For them, both violation types were equally ungrammatical. Therefore we suggest that the P600 for the non-natives reflects highly skilled syntactic processes which, however, are based only on very restricted language input, and therefore do not show the same case specific modulations as the same processes for native speakers. In sum, the learners show similar characteristics in timing of processes elicited by double nominative case violations as native speakers. The differences in the topographical distribution of the effect, however, was taken as an indication that the underlying processes in native and non-native speakers are not identical. Together with the absence of the negativity for double accusative violations, these results were taken to suggest that the learners applied a more formal, though shallower, processing strategy than natives, which seemed to be biased towards the use of the more salient case marker *ga*.

## Conclusion

The application of ERPs to the on-line processing of case violations in a miniature version of Japanese revealed differences between native and non-native speakers' processes despite comparable behavioural performance. The results of Japanese native speakers can be explained best by resorting to syntactic and thematic features of Japanese. While the general N400-P600 pattern indicates similar case processing mechanisms in Japanese as in other free-order case marking languages, for example German, the specific modulation of the P600 component for double accusatives points to the influence of specific syntactic properties of Japanese. In contrast, the results of non-native learners suggest the use of a different, probably shallower, processing strategy which emphasizes phonologically salient case information. This points to the importance of lower level perceptual processes for the efficiency of higher level linguistic processes.

## Methods

### Participants

Two groups of participants were tested, one group of Japanese native speakers and one group of German native speakers trained in Mini-Nihongo. The Japanese native group consisted of 19 right-handed native Japanese speakers (16 female) who were between 20 and 31 years of age (mean: 24.2 years). Four of the Japanese native participants had already taken part in a previous Mini-Nihongo study (cf. [35]). The non-native learner group consisted of 24 German native speakers (12 female) who had already participated in a previous Mini-Nihongo study (cf. [35]) approximately 6 months earlier. All German participants were right handed and were between 20 and 26 years of age (mean: 23.6 years). All participants gave signed informed consent in accordance with the declaration of Helsinki prior to the experiments. The study was approved by the ethics committee of the medical department at the University of Leipzig.

When participants, both trained German and native Japanese participants, had an error rate greater than 40% in at least one violation condition in the ERP experiment, their data were excluded from the analysis in the behavioral and in the ERP data. However, this was the case only for one participant in the non-native group.

### Training

The German participants, who had already received training in the previous Mini-Nihongo study, did a refreshment training. The audio-visual computer learning game comprised comprehension as well as production tasks. Meaning and structure of Mini-Nihongo were (re)learned by trial-and-error learning, which was guided by visual feedback on the computer screen. The sentences were presented auditorily. Individual training times reached from 1 to 3.25 (average: 1.7) hours. All participants reached the final proficiency criterion of 100% in a production task and 75 % in a comprehension task.

### Stimuli

In the ERP experiment participants listened to canonical and non-canonical correct sentences and sentences containing either a double accusative or a double nominative case violation. Examples of sentences of the six conditions are listed in Table 1.

Of the 2048 sentences that can be built in Mini-Nihongo, 64 experimental sentences were chosen for each of the two word orders. The violation stimuli consisted of 64 sentences per condition, and were incorrect variations of the correct sentences. 128 correct and 128 incorrect filler items were added. The total stimuli comprised 512 sentences which had not occurred in the training game. Each verb, noun, classifier and number was repeated with equal frequency. All sentences were spoken by a female native Japanese speaker. The sentences were digitized with a sampling rate of 44 kH and normalized to the same intensity. For experimental presentation the stimuli were divided into eight blocks, each of which contained 64 sentences. Within each block each of the four experimental conditions occurred with equal frequency. Every block was pseudorandomized twice with the constraint that no more than three trials of the same condition could be repeated in sequence. The eight blocks of each of the two randomisations were put in two different orders, which created four lists in total. Those four lists had two different versions varying in the button press configuration (right correct vs. left correct) so that eight versions of stimulus presentation lists resulted in the end.

### Procedure

Before the experiment started, the German participants completed the comprehension test of the learning game in order to assure equal familiarity with the materials among participants (as the last training of the participants varied between 1 and 2 days before the experiment took place). All participants received written instruction in their native language before the ERP experiment. The experimental task was to judge if the sentence they had heard was correct. In order to disentangle effects of motor preparation with linguistic effects the judgment was given 1500 ms after the stimulus offset. During the ERP experiment, participants sat in a comfortable chair in a sound-attenuated booth, 130 cm in front of a computer screen. The 512 sentences were presented via speakers. Participants were asked to fixate at a cross which appeared in each trial in the middle of the screen from 500 before to 1500 ms after stimulus presentation. Then the cross was replaced for up to 2000 ms by two face-like icons appearing on the left and on the right side of the screen, which indicated the configuration of the response buttons. Between trials there was a time interval of 1500 ms. At the end of each block there was a short pause. Participants were instructed to avoid eye movements during sentence presentation.

### Data acquisition

During the experiment, response times and accuracy rates were recorded. Reaction times are not reported here due to the delay between stimulus offset and the response signal, which makes them difficult to interpret. Accuracy rates, however, were an important criterion for the ERP evaluation and therefore will be reported later.

The EEG was recorded from 59 Ag/AgCl electrodes mounted in an elastic cap (Electro Cap International). The vertical electro-oculogram (EOG) was recorded from two electrodes placed above and below the right eye. The horizontal EOG was measured by electrodes placed at the outer canthus of each eye. During recording the EEG was referenced to the left mastoid and afterwards rereferenced to the linked mastoids. Electrode impedances were kept below 5 kO and sampling rate was 250 Hz. Trials containing artefacts due to eye movements, muscular activity or amplifier saturation were excluded from ERP averaging. ERPs were averaged in the time window from -200 to 1500 ms, time-locked to the onset of the noun. The epoch from -200 to 0 ms relative to stimulus onset was taken as an amplitude baseline. An 8 Hz low-pass filter was used for the graphic illustrations only. All statistical evaluations were carried out with unfiltered ERP data.

### Data analysis

For all statistical analyses the SAS 8.2 software package was used. To evaluate differences in accuracy rates on the grammaticality judgment between the Japanese and the trained participants, separate t-tests were calculated for each condition. In the ERP analysis 23.7% of the trials were rejected because of ocular movements or amplifier blocking. Only correctly answered trials were averaged for Japanese and trained participants. After visual inspection two time windows were determined for statistical analysis based on visual inspection and on typical time windows for N400 and P600 effects. Thus, mean amplitudes were calculated for the epoch between 350–500 ms (N400) and between 600 and 900 ms (P600) after onset of the case marked noun.

In order to assess topographic differences of the ERPs, lateral electrodes were summed up in four 'regions of interest' (ROIs) (left-anterior: FP1, AF7, AF3, F7, F5, F3, FT7, FC5, FC3; right-anterior: FP2, AF8, AF4, F8, F6, F4, FT8, FC6, FC4; left-posterior: TP7, CP5, CP3, P7, P5, P3, PO7, PO3, O1; right-posterior: TP8, CP6, CP4, P8, P6, P4, PO8, PO4, O2). As main statistical analysis, a global five-factorial ANOVA was calculated, including the within-subjects factors C (incorrect vs. correct), H (left hemisphere vs. right hemisphere), R (anterior region vs. posterior region) and CA (canonical vs. non-canonical sentences), and the between-subjects factor G (native group vs. non-native group). Further analyses were calculated according to a hierarchical decision criterion. When the global ANOVA revealed a significant interaction (p < .05) including the factor C, additional ANOVAS were calculated to test which effects on the lower levels were driving the interaction (cf. [61,62] for similar procedure).

In order to test if topographical differences between conditions and groups were due to overall amplitude differences, we calculated additional ANOVAS with data from vector-scaled difference waves. For each subject, electrode location, and time window, we calculated difference scores of ERP amplitudes by subtracting the mean amplitudes observed in the correct condition from the corresponding mean scores in the incorrect condition. In order to remove confounding effects of overall amplitude differences between the two groups, each subject's difference scores were normalized by the vector scaling method [63,64]. For each group separately, the across-subjects vector length was computed as RMS amplitude of the grand mean scalp distribution of the difference scores. Then each subject's individual difference scores were divided by the vector length. By this scaling procedure, grand mean amplitude differences were normalized between groups without eliminating within-group, i.e. between-subjects, variability. Since vector scaling was computed over difference scores, distortions due to task-related background activity could be avoided [65-67]. Whenever significant interactions, including a topographical factor, are reported, the significance level of the corresponding effect from the analysis with vector scaled data is reported.

## Authors' contributions

JLM co-designed the study, performed and analysed the experiments, and wrote the first draft of the manuscript, MH co-wrote the first draft of the manuscript and provided psycholinguistic background, ADF co-designed the study and provided psycholinguistic background. All authors read and approved the final manuscript.

## Figures and Tables

**Table 1:** Stimulus examples

*Correct canonical condition*		
Ichi wa no kamo ga 1 [bird] [gen.] duck [nom.]	ni hiki no nezumi o 2 [small-animal] [gen.] mouse [acc.]	tobikoeru tokoro desu. jump over take place.
*Correct non-canonical condition*		
Ichi wa no hato o 1 [bird] [gen.] pigeon [acc.]	ni hiki no neko ga 2 [small-animal] [gen.] cat [nom.]	tobikoeru tokoro desu. jump over take place.
*Double nominative case violation*		
Ichi wa no kamo ga 1 [bird] [gen.] duck [nom.]	ni hiki no nezumi ga 2 [small-animal] [gen.] mouse [nom.]	tobikoeru tokoro desu. jump over take place.
*Double accusative case violation*		
Ichi wa no hato o 1 [bird] [gen.] pigeon [acc.]	ni hiki no neko o 2 [small-animal] [gen.] cat [acc.]	tobikoeru tokoro desu. jump over take

Gen. = genitive, nom. = nominative, acc. = accusative; incorrect elements are underlined.

**Table 2:** Behavioral results

	*native*	*trained non-native*
	
*Correct condition – canonical*	96.3% (SD 3.9)	97.5% (SD 3.6)
*Correct condition – non-canonical*	96.1% (SD 3.8)	96.8% (SD 4.9)
*Case violation – canonical*	97.2% (SD 4.0)	95.2% (SD 5.7)
*Case violation – non-canonical*	97.9% (SD 3.6)	96.3% (SD 5.5)

Mean percentage of accurate grammaticality judgements for native Japanese (n = 19) and trained non-native participants (n = 23) (standard deviation in parentheses).

**Table 3:** Global ANOVAs for unscaled (A) and vector-scaled (B) data.

*A) global ANOVA: 350–500 ms*					*global ANOVA: 600–900 ms*			
*Effect*	*df*	*F value*	*p value*	*MS*_*e*_	*df*	*F value*	*p value*	*MS*_*e*_

C	1,40	26.79	<.0001	4.85	1,40	18.80	<.0001	8.10
C × G	1,40	1.69	.20	4.85	1,40	< 1		
C × CA	1,40	< 1			1,40	1.19	.28	3.32
C × CA × G	1,40	2.39	.13	3.04	1,40	11.96	.001	3.32
C × R	1,40	< 1			1,40	42.33	<.0001	2.05
C × R × G	1,40	< 1			1,40	< 1		
C × R × CA	1,40	< 1			1,40	< 1		
C × R × CA × G	1,40	10.96	<.01	0.90	1,40	< 1		
C × H	1,40	2.43	.13	0.42	1,40	1.83	.18	0.27
C × H × G	1,40	< 1			1,40	< 1		
C × H × CA	1,40	3.83	.06	0.57	1,40	< 1		
C × H × CA × G	1,40	< 1			1,40	< 1		
C × R × H	1,40	1.29	.26	0.13	1,40	< 1		
C × R × H × G	1,40	< 1			1,40	< 1		
C × R × H × CA	1,40	3.34	.08	0.08	1,40	5.11	<.05	0.17
C × R × H × CA × G	1,40	< 1			1,40	5.47	<.05	0.17

*B) global ANOVA: 350–500 ms*					*global ANOVA: 600–900 ms*			

*Effect*	*df*	*F value*	*p value*	*MS*_*e*_	*df*	*F value*	*p value*	*MS*_*e*_

G	1,40	< 1			1,40	< 1		
CA	1,40	1.54	.22	0.08	1,40	< 1		
CA × G	1,40	< 1			1,40	8.84	<.01	0.01
R	1,40	< 1			1,40	44.99	<.0001	0.01
R × G	1,40	< 1			1,40	< 1		
R × CA	1,40	< 1			1,40	< 1		
R × CA × G	1,40	7.78	<.01	0.01	1,40	< 1		
H	1,40	1.78	.19	0.01	1,40	1.00	.32	0.01
H × G	1,40	< 1			1,40	< 1		
H × CA	1,40	3.66	.06	0.01	1,40	< 1		
H × CA × G	1,40	< 1			1,40	< 1		
R × H	1,40	< 1			1,40	1.50	.22	0.003
R × H × G	1,40	< 1			1,40	< 1		
R × H × CA	1,40	3.85	.06	0.001	1,40	6.42	<.05	0.002
R × H × CA × G	1,40	< 1			1,40	11.06	<.01	0.002

In the analysis with unscaled data, mean amplitude averages for single conditions were included in a five-factorial ANOVA with four within-subjects factors (C, CA, R, H) and one between-subjects factor (G). For the analysis with scaled data, difference waves were calculated by subtracting mean amplitude averages of the correct condition from the averages of the incorrect condition. The four-factorial ANOVA for scaled data included the within-subject factors (CA, R, H) and the between-subjects factor G. G = Group (native/non-native); C = Condition (correct/incorrect); CA = Case (canonical/non-canonical); R = Region (anterior/posterior); H = Hemisphere (left/right).
